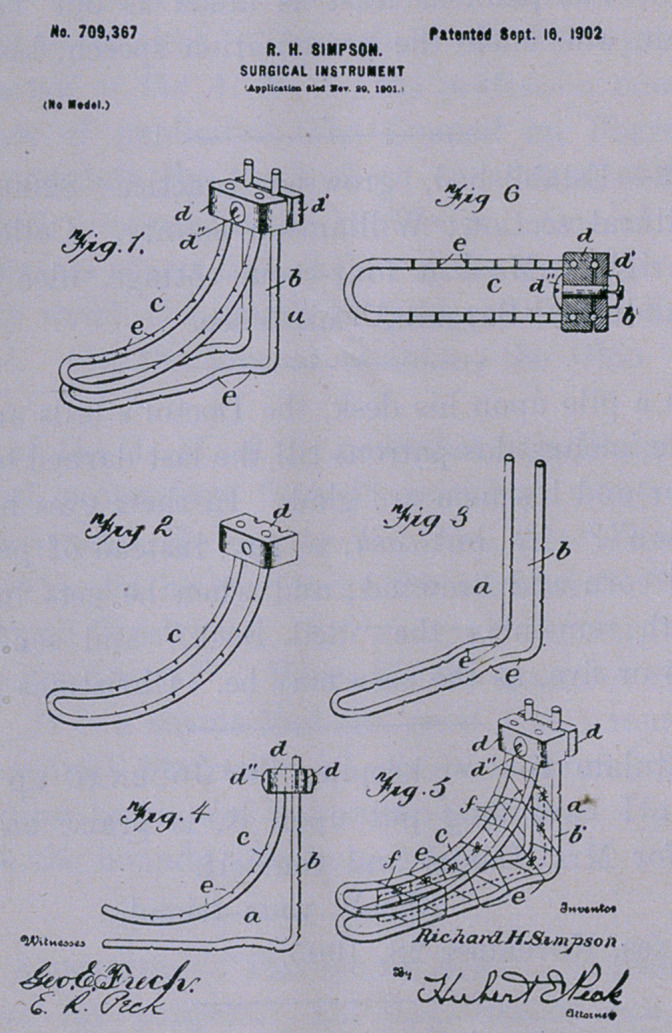# Editorialets

**Published:** 1905-12

**Authors:** 


### Editorialets.

---

MERRY CHRISTMAS, DOCTOR! And a Happy New Year!

---

THE SEVENTH COUNCILOR DISTRICT MEDICAL SOCIETY will meet in Austin, Thursday, December 21st inst.

---

The citizens of Memphis made up a purse of $10,000 and presented it to Dr. Heber Jones, the health officer, for *doing his duty* (for which he was paid, at least as much as our Tabor), and a pretty woman, who made the presentation speech, kissed him, besides.

---

FOR SALE.—Established, growing practice—$2000 to $2500. Rich agricultural section; Williamson county. Collections good; competition right. Modern four-room cottage, nice barn; $1500 will buy. Address, "Bargain," this office.

---

Heaped in a pile upon his desk, the Doctor's bills are laid;
He'll rustle 'mongst his patrons till the last darned one is paid.
The farmer and his men are glad. In their eyes he sees
"Speculation"? No, but *cash,* to pay, instead of *peas,*
and hay and corn and firewood; and when he gets in his bills he will forthwith remember the "Red Back," and send along that dollar or two or five, as the case may be. Christmas gift, Doctor!

---

To congratulate you on keeping the JOURNAL up to the high estimate that I have long put upon it, is praise enough. With best wishes for Mrs. Daniel and yourself.

Truly your friend,

Dallas, Texas, November 28, 1905. S. EGAN.

---

A RARE OPPORTUNITY is offered to secure a $2500 practice and a beautiful home and small farm in a middle Texas county, in the richest farming and cattle section of the State. The property consists of well improved 60 acres in a thriving village, 30 in cultivation and 30 divided in two pastures of fine Bermuda grass, a nice five-room cottage, with bath and all other conveniences, large barn and other outhouses, abundant water, well and windmill and private waterworks. Forty-eight acres will be sold separately if desired, and the 12 acres with the improvements is offered for $2000, or the whole for $3500. This is a real bargain and a rare opportunity to step into a paying practice in a splendid section of country and a prompt paying community. Terms on request. Dr. C. F. C., care this Journal, Austin, Texas.

---

YOUR GRANDFATHER WAS NOT TO BLAME. Why? See Adv. Burnham S. I. Co.

---

PATENT FOR SALE.—The patent of a new surgical instrument, illustrated below, to aid in the operation of perineorrhaphy, is for sale.

The broad object of this invention is to provide an instrument or frame-work whereby the lacerated edges can be brought accurately together by sutures, and be thus held in position without suture or pressure until union is complete. The instrument will fill a long-felt want, and the physician will readily consider the attitude he is in in case of laceration; the grievous condition of the woman if union is not perfect and the obstacles he has to combat with the means now at his command.

For price and terms, address Dr. Daniel, Austin, Texas, or the inventor, Dr. R. H. Simpson, Turnersville, Texas.

Instrument for Perineorrhaphy. Invented and patented by R. H. Simpson, M. D., Turnersville, Texas.

---

FOR SALE.—My home and practice. Home consists of a 6-acre lot, one-half in Bermuda grass, the other with residence and outbuildings. Located in Thornton, on the H. & T. C. R. R., Limestone county, Texas. If you want to secure a good practice, write me at once. Terms cash. T. M. Wilson, M. D.

---

*The Medical and Surgical Monitor* and the *Central States Medical* have amalgamated under the name of the *Central States Medical Monitor,* Indianapolis, Ind. Dr. S. E. Earp, formerly editor of the *Central States* will be editor, and Dr. S. P. Scherer, formerly editor of the *Monitor,* will be associate editor.

---

EVERY LITTLE BIT HELPS.—"One of the spicest little journals that comes to the Secretary is the *American Medical Journalist.* It contains many very readable articles, and its last issue seems to be chiefly directed against the various transactions of the American Medican Association, especially against the *Journal. We rather think that there is a great deal of truth in what it has to say and advise all who wish to know both sides of the question to read it.* It is published by D. A. O'Gorman, of New York."—From the *Journal of the South Carolina Medical Association* (published under the direction of the Publication Committee of the South Carolina Medical Association), Charleston, S. C., September 21, 1905.

## Figures and Tables

**Fig.1. Fig 2 Fig 3 Fig. 4 Fig.5. Fig 6 f1:**